# Role of tumor-associated macrophages in hepatocellular carcinoma: impact, mechanism, and therapy

**DOI:** 10.3389/fimmu.2024.1429812

**Published:** 2024-08-07

**Authors:** Yinqi Zhang, Guoyong Han, Jian Gu, Zhiqiang Chen, Jindao Wu

**Affiliations:** ^1^ Hepatobiliary Center, The First Affiliated Hospital of Nanjing Medical University, Nanjing, Jiangsu, China; ^2^ Key Laboratory of Liver Transplantation, Chinese Academy of Medical Sciences, Nanjing, Jiangsu, China; ^3^ National Health Commission (NHC) Key Laboratory of Hepatobiliary Cancers, Nanjing, Jiangsu, China

**Keywords:** hepatocellular carcinoma, tumor-associated macrophages, tumor microenvironment, treatment resistance, tumor angiogenesis, immunotherapy

## Abstract

Hepatocellular carcinoma (HCC) is a highly frequent malignancy worldwide. The occurrence and progression of HCC is a complex process closely related to the polarization of tumor-associated macrophages (TAMs) in the tumor microenvironment (TME). The polarization of TAMs is affected by a variety of signaling pathways and surrounding cells. Evidence has shown that TAMs play a crucial role in HCC, through its interaction with other immune cells in the TME. This review summarizes the origin and phenotypic polarization of TAMs, their potential impacts on HCC, and their mechanisms and potential targets for HCC immunotherapy.

## 1 Introduction

Primary liver cancer, including hepatocellular carcinoma (HCC) (comprising 75%–85% of cases) and intrahepatic cholangiocarcinoma (10%–15%), as well as other rare types, is one of the most frequent malignancies worldwide, with global morbidity and cancer‐related mortality ranking sixth and third, respectively (1). In Asia, liver cancer is the fifth most common cancer and the second leading cause of malignant death. HCC, which is the most common histological type, accounts for the majority of incidence and mortality of liver cancer cases (2). The main risk factors for HCC are chronic infection with hepatitis B virus (HBV) or hepatitis C virus (HCV), aflatoxin-contaminated foods, heavy alcohol intake, excess body weight, type 2 diabetes, and smoking (3). Although emerging treatments such as immunotherapies targeting the programmed death receptor 1 (PD-1) or its ligand (PD-L1) have been approved for the treatment of HCC with a major effect on patient survival (4), still there are patients who cannot benefit from them. The high incidence and mortality of liver cancer place a heavy burden on patients, economically and mentally.

The tumor microenvironment (TME), placing great emphasis on tumorigenesis, progression, and metastasis toward HCC, also strongly contributes to the tolerogenic immune response of HCC treatment (5, 6). It comprises and can be affected by multiple components including tumor-associated macrophages (TAMs), tumor-associated neutrophils (TANs), cancer-associated fibroblasts (CAFs), myeloid-derived suppressor cells (MDSCs), and regulatory T cells (Tregs) (7, 8). TAMs, which are those macrophages infiltrating the TME, not only have an impact on the suppression of antitumor immune responses but also contribute to tumor immune surveillance and antitumor responses (9–12). Due to the key role that TAMs play in HCC, hepatic macrophages have long been considered as potential therapeutic targets for various HCC treatment modalities. A better understanding of the impact and mechanism of TAMs in regulating HCC tumorigenesis, progression, and metastasis is essential for the improvement of immunotherapy (13).

In this review, we summarize the origin and phenotypic polarization of TAMs, their potential impacts on HCC, and their mechanisms and potential targets for HCC immunotherapy.

## 2 Origin and phenotypic polarization of TAMs

According to the origin of liver macrophages, they can be classified into two types: tissue-resident macrophages, also known as Kupffer cells (KCs), and monocyte-derived macrophages (14). KCs, which are abundant in normal liver tissue, are developed from erythromyeloid progenitors (EMPs) in the yolk sac or fetal liver (15). In the progression of liver cancer, multiple protumorigenic factors would force KCs to recruit immune cells including the number of monocytes in the liver to modulate inflammation and prompt the functional differentiation of KCs since they are immunogenic in nature (16, 17). Those macrophages continue infiltrating tumors and eventually differentiate into TAMs (18).

**Figure 1:**
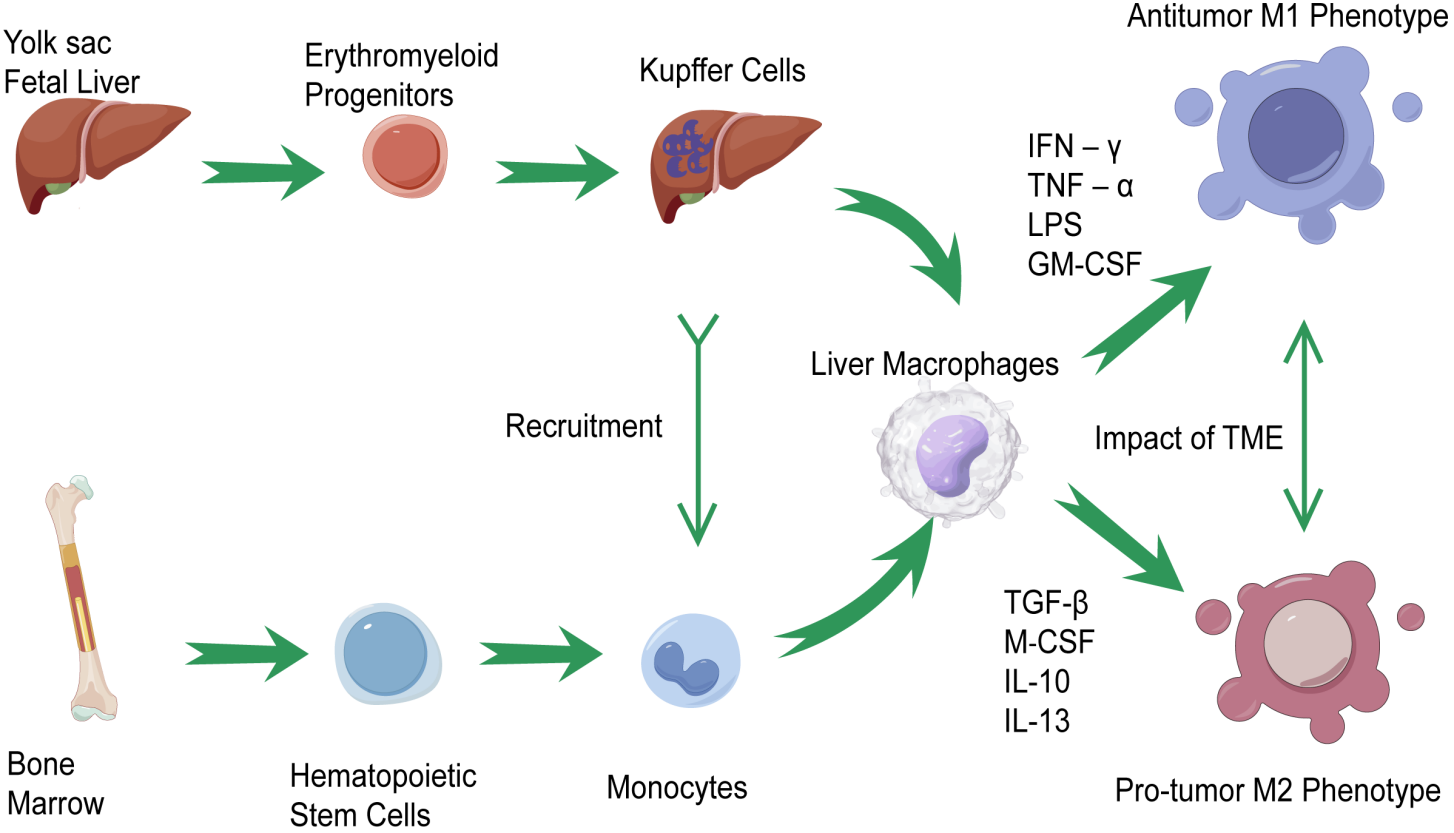
The origin and phenotypic polarization of tumor-associated macrophages (TAMs) in hepatocellular carcinoma (HCC). Kupffer cells (KCs) and monocyte-derived macrophages are the two classifications of liver macrophages. Kupffer cells, residing in tissues, are differentiated from erythromyeloid progenitors (EMPs) in yolk sac or fetal liver. Under the recruitment of KCs, monocyte-derived macrophages modulate inflammation in the progression of liver cancer and prompt the functional differentiation of KCs, which continue infiltrating tumors and differentiate into TAMs. TAMs would undergo M1-like activations through the stimulation of interferon‐γ (IFN‐γ), tumor necrosis factor‐α (TNF‐α), lipopolysaccharide (LPS), and granulocyte-macrophage colony-stimulating factor (GM-CSF). On the other hand, the M2-like activations of TAMs are induced by transforming growth factor (TGF)-β, macrophage colony-stimulating factor (M‐CSF), IL-10, and IL-13. Under the impact of the tumor microenvironment (TME), the polarization of TAMs is joined in a dynamic cycle.

The macrophage polarization theory indicates that TAMs undergo M1-like or M2-like activation and are divided into two types that have contrasting functions: the antitumor M1 phenotype and the protumor M2 phenotype (19, 20). M1-like macrophages are induced by interferon‐γ (IFN‐γ), tumor necrosis factor‐α (TNF‐α), lipopolysaccharide (LPS), and granulocyte-macrophage colony-stimulating factor (GM-CSF) (21). Because of their ability in antigen presentation, M1-like macrophages could promote the recruitment of type 1 helper T (Th) cells to enhance antitumor responses, kill tumor cells, and suppress tumors (19, 22, 23). M2-like macrophages are induced by transforming growth factor (TGF)-β, macrophage colony-stimulating factor (M‐CSF), interleukin (IL)-10, and IL-13 (24, 25). Under the influence of those cytokines, M2-like macrophages suppress effector T-cell infiltration, activate Th2-type immune responses, and promote the progression of tumor (19, 26). It should be pointed out that the polarization of macrophages is joined in a dynamic cycle under the impact of the TME (27–29). More importantly, the M1-like/M2-like dichotomy based on *in-vitro* experiments may be defective because of the high plasticity of TAMs in the TME. Increasing evidence based on single-cell RNA sequencing (scRNA-Seq) has revealed that an M1-like/M2-like paradigm could not classify the complex phenotype of TAMs precisely, and a higher resolution than M1-like/M2-like is therefore required to categorize the molecular signatures of TAM subtypes in the TME (30, 31). Taking all these factors into consideration, TAMs, as a potential target in HCC immunotherapy, should be accorded great importance. It is essential for us to understand the role and function of TAMs in HCC and develop novel immunotherapies. **Figure 1** summarizes the origin and phenotypic polarization of TAMs in HCC.

## 3 Impact of TAMs on HCC and their mechanism

**Figure 2:**
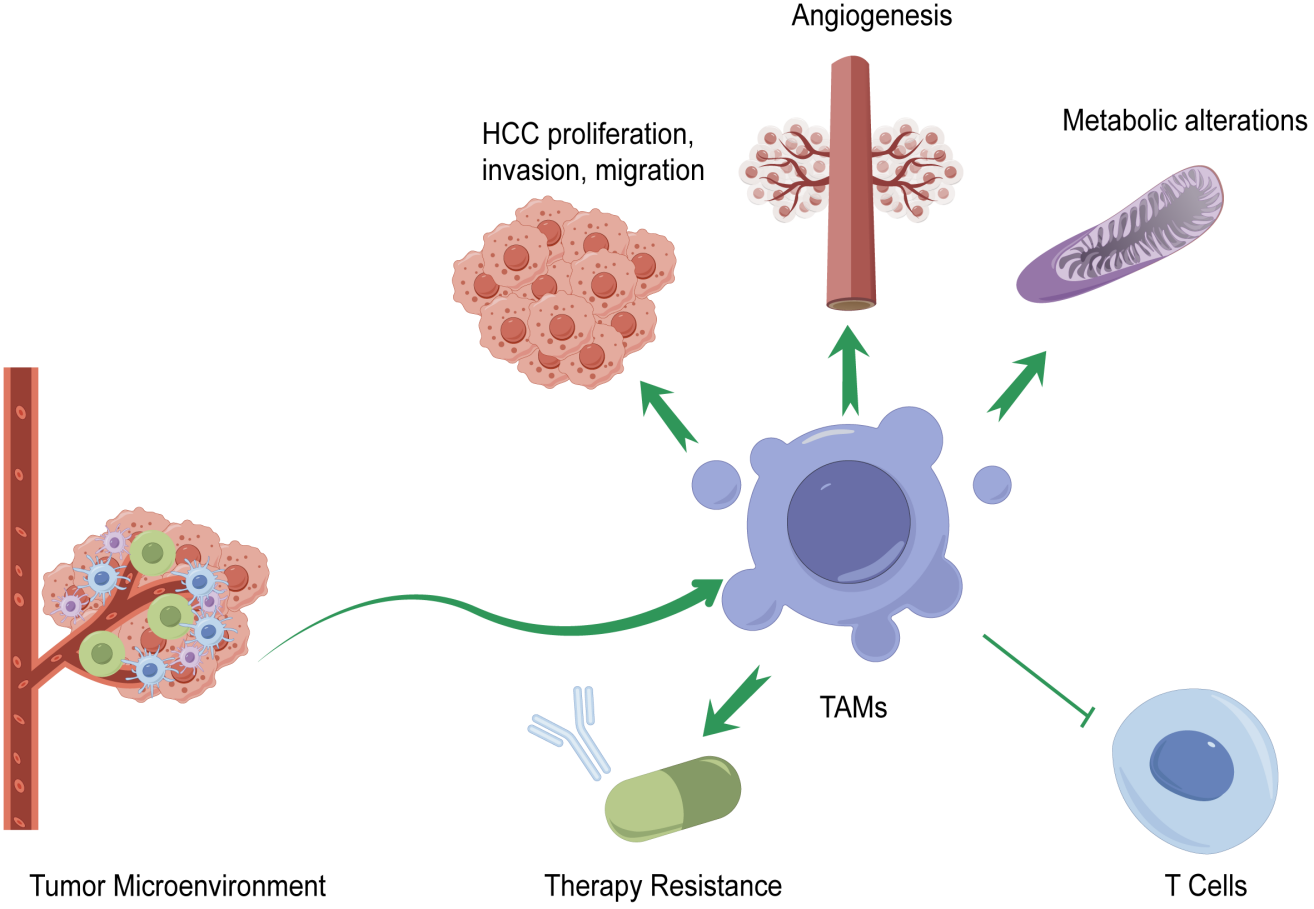
Mechanisms of TAMs in the pathogenesis of HCC. TAMs affect the progression of HCC by promoting or suppressing HCC proliferation, invasion, and migration; generating immune checkpoint blockade (ICB) therapy resistance; affecting HCC angiogenesis; regulating immune functions of various types of T cells; and altering metabolism.

TAMs are regulated by multiple factors in the TME of HCC. The infiltration of TAMs in HCC is related to HCC progression, therapy resistance, tumor angiogenesis, immunity, and metabolic alterations. The mechanisms of TAMs in the pathogenesis of HCC are summarized in **Figure 2**.

### 3.1 Regulation of the phenotypic polarization and infiltration of TAMs in HCC

In HCC, M1-like macrophages represent anticancer characteristics, which can suppress tumor progression through various mechanisms, while M2-like macrophages which are enriched in HCC tissue, according to The Cancer Genome Atlas Program (TCGA), are regarded as a protumoral type (19, 32). Liu N et al. identified that M2 polarization of KCs impairs hepatic enrichment of CD8^+^ T cells, while microRNA (miR)-206 drives M1 polarization of KCs and hepatic recruitment of CD8^+^ T cells through C-C motif chemokine ligand 2 (CCL2) production (33). The high expression of retinoic acid-inducible gene I (RIG-I) and sirtuin1 (SIRT1) in HCC regulates M1 polarization via the nuclear factor kappa-light-chain-enhancer of activated B-cell (NF-κB) pathway (34, 35). Studies conducted by Zhang Y et al. revealed that matricellular protein spondin2 (SPON2) and its integrin receptor α4β1 facilitate M1-like macrophage recruitment to the TME to prevent HCC progression (36). In addition, Wang Q et al. proved that IL-12-overexpressed monocytes could directionally differentiate into M1-like macrophages through downregulation of the signal transducer and activator of transcription (STAT) 3 and result in the inhibition of HCC growth (37).

M2-like macrophages could be divided into four subtypes based on their stimulant factors: M2a, which is induced by Th2 cytokines; M2b, which is induced by immune complexes; M2c, which is induced by anti-inflammatory cytokines or glucocorticoids; and M2d, which is induced by IL-6-like cytokines (38). Tumor acidosis could trigger regulatory macrophages and enhance immune evasion (39), which eventually causes the generation of macrophages with immunosuppressive properties. Tan S et al. proved that zinc-fingers and homeoboxes 2 (Zhx2) bind to p65 protein and regulate NF-κB activation, while lactate domesticates macrophages through transcriptional regulation of Zhx2, reduces Zhx2 expression in TAMs and, in turn, attenuates the immunogenic M1-like activation of macrophage, increases the polarization of M2-like macrophages induced by IL-4, and fosters the liver tumor progression in an NF-κB–Irf1-dependent manner (40). Lu Y et al. identified that loss of xanthine oxidoreductase (XOR) increases α-ketoglutarate generation in monocyte-derived TAMs by increasing the activity of isocitrate dehydrogenase 3α (IDH3α) and drives macrophage differentiation toward the M2 phenotype (41). Wang Y et al. found that the circular RNA (circRNA) hsa_circ_0074854 is related to exosome-mediated M2-like macrophage polarization (42). Yin C et al. found that HCC cell-secreted miR-146a-5p could be delivered by exosomes into macrophages and promote macrophages toward M2-like polarization (43). Yang Y et al. demonstrated that HCC cell-derived Wnt ligands via Wnt/β-catenin signaling promote M2-like macrophage polarization (44). In macrophages, Wnt/β-catenin signaling can be activated by the long non-coding RNA (lncRNA) LINC00662 through WNT family member 3A (Wnt3a) secretion in a paracrine manner and further promoted M2-like macrophage polarization (45). Chen M et al. proved that T follicular helper (T_FH_) cells operate via the IL-21–IFN-γ pathways to induce plasma cells and create conditions for M2b macrophage polarization, while T_FH_ cell induction is based on Toll-like receptor (TLR) 4-mediated monocyte inflammation and subsequent T-cell STAT1 and STAT3 activation (46).

### 3.2 TAMs affect HCC proliferation, invasion, and migration

A growing number of studies and lines of evidence have shown that TAMs are related to HCC proliferation, invasion, and migration. In the TME, TAMs and tumor cells interact through mediators such as TGF-β, vascular endothelial growth factor (VEGF), platelet-derived growth factor (PDGF), M-CSF, IL-10, chemokine C-X-C motif ligand (CXCL), and extracellular vesicles (EVs) to affect tumor progression (47). For example, Gunassekaran G et al. demonstrated that IL-4R-Exo(si/mi) inhibits tumor growth by reprogramming TAMs into M1-like macrophages and increasing antitumor immunity (48). Xu M et al. found that TAMs augment the aerobic glycolysis in HCC cells and their proliferation by the extracellular exosome transmission of a myeloid-derived lncRNA, M2 macrophage polarization-associated lncRNA (lncMMPA), which could not only polarize M2 macrophage but also act as a microRNA (miRNA) sponge to interact with miR-548 and increase the mRNA level of aldehyde dehydrogenase 1 family member A3 (ALDH1A3) (49). TAMs could contribute to tumor development by inducing the expression of hepatocyte growth factor (HGF) (50). Wan S et al. and Mano Y et al. proved that TAMs could release IL-6 to enhance the expansion of human HCC stem cells, participate in tumorigenesis, and promote HCC progression via the STAT3 signaling pathway (51, 52). M2-like macrophages could be induced by HCC-derived IL-8 and promote a pro-oncogenic inflammatory microenvironment, which would directly promote epithelial–mesenchymal transition (EMT) of HCC cells and stimulate their invasive potential (53). M2-like macrophages are also considered to promote HCC migration via the TLR4/STAT3 signaling pathway (54). Despite M1-like macrophages being thought to be tumoricidal, Zong Z et al. proved that M1-like macrophages secreted IL-1β to induce PD-L1 expression through the transcription factors interferon regulatory factor 1 (IRF1) and NF-κB in HCC cells, supporting the protumor progression role of M1 macrophages (55).

miRNAs are small non-coding molecules that can regulate gene expression at the post-transcriptional level and exhibit important regulatory roles in mediating the effects of TAMs on HCC progression. It has been proven that miR-23a-3p, highly expressed in M2 TAM-derived exosomes, enhances HCC metastasis by targeting phosphatase and tensin homolog (PTEN) and tight junction protein 1 (TJP1) (56). MiR-146a-5p, enriched in HCC exosomes, can be regulated by the transcription factor Sal-like protein-4 (SALL4) and is demonstrated to promote infiltration of M2 TAMs, which results in T-cell exhaustion and HCC progression (43). On the other hand, MiR-148b deficiency promoted HCC growth and metastasis through colony-stimulating factor 1 (CSF1)/CSF1 receptor (CSF1R)-mediated TAM infiltration (57). Ning J et al. found that the miR-17–92 cluster, originating from the extracellular EVs of M2-like macrophages, stimulated the imbalance of TGF-β1/BMP-7 pathways in HCC cells by inducing TGF-β type II receptor (TGFBR2) post-transcriptional silencing and inhibiting activin A receptor type 1 (ACVR1) post-translational ubiquitylation by targeting Smad ubiquitylation regulatory factor 1 (Smurf1), thus improving HCC cell growth and metastasis (58). Zhang J et al. demonstrated that TAM-derived prostaglandin E2 (PGE2) stimulates ubiquitin-like, containing PHD and RING finger domains 1 (UHRF1) expression by repressing miR-520d that targets the 3′-UTR of UHRF1 mRNA, while UHRF1 induces DNA hypomethylation of the CSF1 promoter and promotes CSF1 expression, thereby leading to TAM recruitment and activation which sustains PGE2 production in a self-enhancing oncogenic microenvironment to improve HCC progression (59). On the other hand, Wang L et al. proved that miR-628–5p, derived from M1-like macrophages, could inhibit the m6A modification of circFUT8, inhibiting HCC development (60).

Recently, Wu L et al. identified a 500-µm-wide zone centered around the tumor border in patients with liver cancer through nanoscale resolution-SpaTial Enhanced Resolution Omics-sequencing (Stereo-seq), referred to as “the invasive zone,” where overexpression of CXCL6 could induce activation of the JAK–STAT3 pathway, which causes SAAs’ overexpression and leads to the recruitment of macrophages and M2-like polarization, resulting in the formation of a local immunosuppressive microenvironment and the promotion of HCC invasion and migration (61).

### 3.3 Impact of TAMs on resistance to HCC treatment

Following the Barcelona Clinic Liver Cancer (BCLC) staging system, those with advanced-stage HCC tumors will first receive systemic therapies (62, 63). Although systemic therapies have substantially improved the reported natural history of untreated cases at advanced-stage HCC, with median survival times of ~6 months in patients with well-preserved liver function defined as Child–Pugh A (according to the Child–Pugh score) and compensated disease (64–66), there remains a large number of HCC patients that do not respond to the treatments. Therefore, uncovering the mechanism of drug resistance and increasing the sensitivity of those drugs will be of great benefit to further improve the overall survival (OS) of patients with HCC. TAMs have been demonstrated to affect immune checkpoint blockade (ICB) therapy, especially with antibodies against the PD-1/PD-L1 signal (67). At the cellular level, an increased concentration of extracellular adenosine as well as the depletion of tryptophan and uncontrolled activation of the PI3K/AKT pathway induces an immune-tolerant TME, reducing the response to immune checkpoint inhibitors (ICIs) (68). Tan J et al. found that the number of triggering receptors expressed on myeloid cell (TREM)-2^+^ TAMs is increased in post-transarterial chemoembolization (TACE) HCC, causing increased Gal-1 secretion to mediate the overexpression of PD-L1 in vessel endothelial cells, which turns out to compromise both the number and function of CD8^+^ T cells and suppress the therapeutic efficacy of anti-PD-L1 blockade (69). Wei C et al. found that protein kinase C alpha (PKCα) phosphorylates zinc finger protein 64 (ZFP64) at S226 and promotes its nuclear translocation, thereby transcriptionally activating CSF1, which further induces the recruitment and M2-like polarization of macrophages, inducing immune escape and anti-PD-1 resistance in HCC (70). Lu J et al. revealed that enhanced expression of CD39 on TAMs, which is induced by HCC-secreted exosomal circTMEM181, could collaborate with CD73 to fulfill the sequential activation of the ATP–adenosine pathway, impair CD8^+^ T-cell function, and build a PD-1 antibody-resistant tumor environment (71). M2-like macrophages are also reported to mediate sorafenib resistance in HCC by secreting HGF (72). On the other hand, TAMs are reported to cause oxaliplatin-based chemotherapy resistance by triggering autophagy and apoptosis evasion in HCC tumor cells (73). To overcome the resistance of TAMs to HCC treatment, research has been carried out to enhance the sensibility of anti-PD-1 therapy in HCC. Wang J et al. found that blockage of Calcyclin-Binding Protein (CacyBP) would inhibit the expression of C-X3-C motif chemokine ligand 1 (CX3CL1), a key chemotactic factor for the recruitment of monocyte-derived macrophages to the liver (74), and thus significantly reduce TAM infiltration and achieve synergies with anti-PD-1 treatment in HCC (75).

### 3.4 TAMs affect angiogenesis in HCC

Vasculature induction is regarded as one of the 14 hallmarks of tumor development (76). The hypervascular nature of most HCC tumors underlines the importance of angiogenesis in the pathobiology of HCC (77). The density of the tumor microvessel is positively correlated with macrophage counts, indicating the key role that TAMs play in HCC angiogenesis (78). Therefore, it is essential to understand the mechanism of TAMs affecting angiogenesis in HCC. MiR-223, a well-documented myeloid-enriched miRNA expressed in neutrophils, macrophages, and hepatocytes, is reported to attenuate hepatocarcinogenesis by blocking hypoxia-driven angiogenesis and immunosuppression (79). Bartneck M et al. found that C-C chemokine receptor type 2^+^ (CCR2^+^) TAMs are enriched in highly vascularized HCC, especially those that arise in fibrotic or cirrhotic livers, and could promote angiogenesis and tumor vascularization in those livers (80). Zang M et al. found that CD14^+^ inflammatory macrophages in HCC tissues could alter macrophage function through persistent IL-23 generation, which are related to the higher concentrations of VEGF and the promotion of HCC development after chronic HBV infection (81). Meng Y et al. identified that the expression of C-X-C motif chemokine receptor 4 (CXCR4), a novel vascular marker for vessel sprouting in HCC tissues, can be promoted by monocytes/macrophages via the ERK pathway in hepatocellular carcinoma (82). On the other hand, combining zoledronic acid (ZA) with sorafenib could improve the antitumor efficacy by downregulating the expression of CXCR4 (83).

### 3.5 TAMs affect immunity in the TME of HCC

Macrophages are closely related to the immune evasion of HCC through expressing a series of immunosuppressing molecules including cytokines, chemokines, and enzymes (84). The interaction between TAMs and CD8^+^ T cells produced an immunosuppressive microenvironment in HCC. Wu Q et al. found that hypoxia-inducible factor 1α (HIF-1α) induced increased expression of TREM-1 in TAMs, resulting in the impairment of the cytotoxic functions of CD8^+^ T cells and the induction of CD8^+^ T-cell apoptosis (85). On the other hand, Xiong H et al. demonstrated that increased IFN-γ signaling following anti-PD-L1 treatment can decrease Arginase-I (ARG1) expression and remodel the macrophage compartment by polarizing it toward a more proinflammatory phenotype to enhance T-cell responses (86). Liao J et al. revealed that a low dose of type I interferon could effectively reprogram human monocyte-derived macrophages to upregulate CD169 expression, and such induced CD169^+^ macrophages exhibited significantly enhanced phagocytotic and CD8^+^ T-cell-activating capacities (87). TAMs can also cooperate with Tregs in suppressing immunity in the TME of HCC (85). In addition, activated and exhausted mucosal-associated invariant T cells (MAITs), represented as an abundant innate-like T-cell subtype in the human liver, have been proven to be associated with disease progression and poor outcomes in HCC patients (88). Ruf B et al. demonstrated that human hepatic CD163^+^ macrophages inhibit liver MAIT cell function through a cell-contact and PD-L1-dependent mechanism (89). Finally, Cheng K et al. proposed that since M2-like macrophages, Tregs, and MDSCs are the main components of the immunosuppressive microenvironment, eliminating TAMs may lead to the compensatory emergence of other protumorigenic immune cells (90).

### 3.6 Metabolic alterations of TAMs in HCC

The tumor progression of HCC is closely related to the alterations of metabolic enzymes, metabolites, and metabolic pathways in macrophages (91, 92). TAMs actively take up and metabolize glucose to acquire immunosuppressive and protumor functions (93). Shi Q et al. revealed that the TME endowed M2-like TAMs with a high capability of glucose uptake and utilization, which enhanced the activity of the hexosamine biosynthetic pathway to enhance O-GlcNAcylation on cathepsin B (CTSB) in TAMs, leading to an elevated mature form of CTSB and its secretion in the TME, which in turn promote tumor metastasis and chemoresistance (94). On the other hand, fatty acid binding protein 5 (FABP5), a lipid-binding protein, could promote macrophage lipid accumulation and foster immune tolerance formation in HCC (95). Wu L et al. found that downregulation of receptor-interacting protein kinase 3 (RIPK3) in the TAMs of HCC facilitated fatty acid metabolism, including fatty acid oxidation (FAO), and induced M2 polarization in the TME (96). Zhang Q et al. found that FAO contributes to IL-1β secretion in M2-like macrophages, which could promote HCC cell migration (97).

## 4 TAMs in HCC immunotherapy

**Figure 3:**
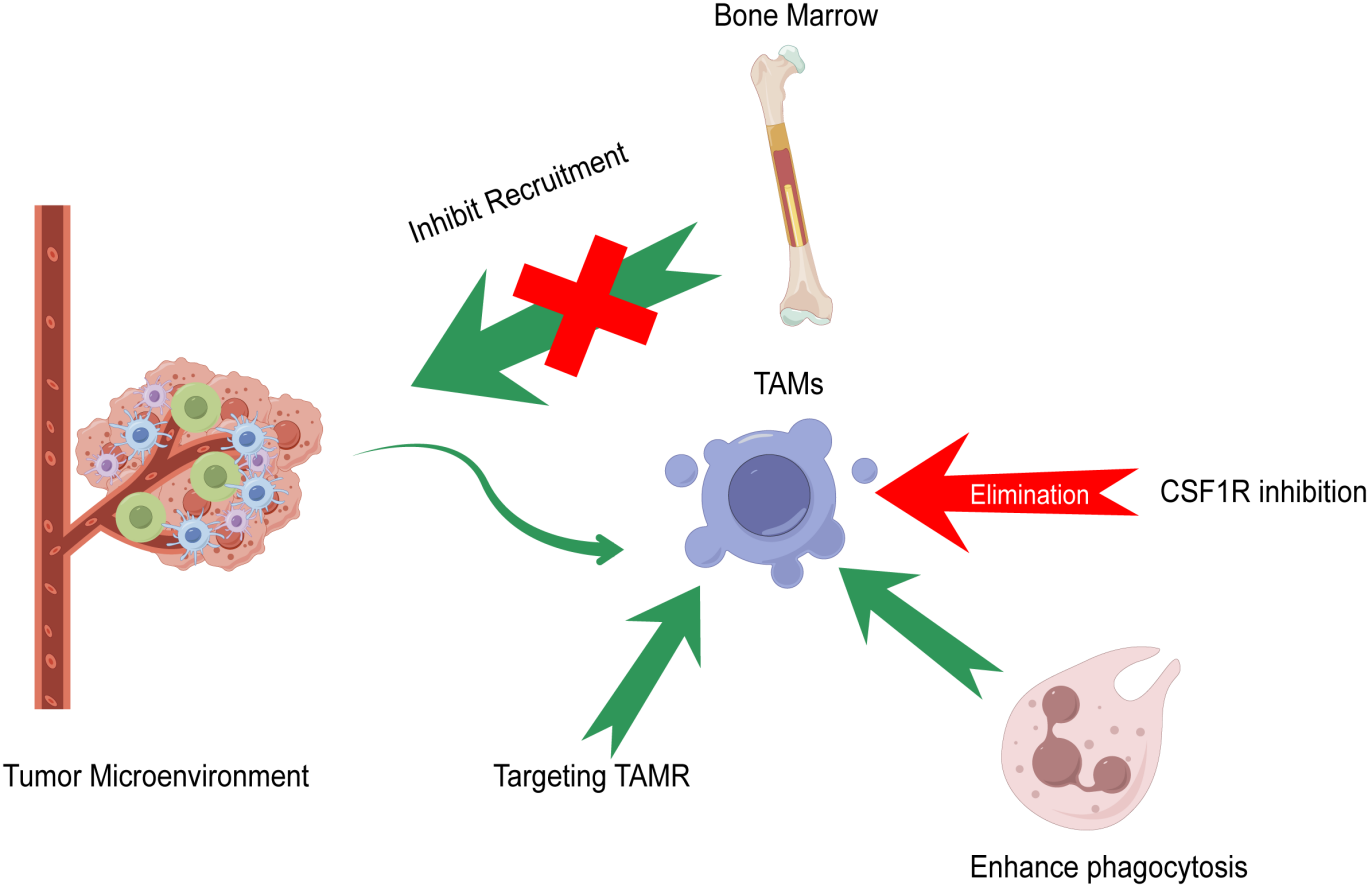
Current strategies of macrophage-targeting therapies. Elimination of TAMs in tumor tissues, inhibition of TAM recruitment, promotion of TAM phagocytosis, and targeting TAM receptors (TAMR) including Tyro3, Axl, and MerTK compose the main strategies of macrophage-targeting therapies.

Immunotherapy is the first‐line treatment for the comprehensive therapy of patients with advanced HCC in China, including atezolizumab combined with bevacizumab, sintilimab combined with a bevacizumab analog, donafenib, rovatinib, and sorafenib. Currently, the four therapeutic strategies targeting TAMs are the elimination of TAMs in tumor tissues, inhibition of TAM recruitment, promotion of TAM phagocytosis, and targeting TAM receptors (TAMRs), including Tyro3, Axl, and MerTK (98). **Figure 3** summarizes the current strategies of macrophage-targeting therapies. **Table 1** summarizes the preclinical studies and clinical trials that focus on macrophage-targeting therapies.

**Table 1:** Studies and undergoing clinical trials of drugs targeting TAMs for HCC treatments.

Study or clinical trial number	Treatment strategy	Drug name	Results
Zhu Y et al. (99)	CSF1R inhibitor	PLX3397	Blocking CSF1/CSF1R enhances the efficacy of immune checkpoint inhibitors for the treatment of HCC.
NCT04050462	Blocking CSF1/CSF1R	Cabiralizumab	N/A
NCT03245190	Blocking CSF1/CSF1R	Chiauranib	N/A
Ambade A et al. (100)	CCL2/CCR5 antagonist	Cenicriviroc	Ameliorates alcohol-induced steatohepatitis and liver damage
NCT04123379	CCL2/CCR5 antagonist	BMS‐813160	N/A
Yao W et al. (101)	CCR2 antagonist	747	Potentiates the therapeutic effect of sorafenib
Li X et al. (102)	CCR2 antagonist	RDC018	Blockade of CCL2/CCR2 signaling suppresses murine liver tumor growth.
Chen J et al. (103)	CD47‐SIRPα blocking	Anti‐CD47‐Ab	Anti-CD47 antibody treatment enhances the curative effect of TACE.
Lo J et al. (104)	CD47‐SIRPα blocking	Anti‐CD47‐Ab	Anti-CD47 antibody treatment enhances the curative effect of doxorubicin.
Xiao Z et al. (105)	CD47‐SIRPα blocking	CD47mAb	CD47mAb enhances the phagocytosis ability of macrophages.

Colony-stimulating factor 1 receptor (CSF1R)-mediated signaling is crucial for the differentiation and survival of the mononuclear phagocyte system, especially macrophages (106). The intratumoral presence of CSF1R^+^ macrophages is related to poor survival in various tumor types (107). Zhu Y et al. found that blocking CSF1/CSF1R could prevent TAM trafficking and thereby enhance the efficacy of immune checkpoint inhibitors for the treatment of HCC (99). Drugs that focus on CSF1R inhibition include RG7155 and IMC-CS4 (108, 109). On the other hand, research has found that specific targeting of CD163^+^ TAMs, a type of M2-like macrophages, could re-educate the tumor immune microenvironment and promote both myeloid and T-cell-mediated antitumor immunity, which illustrates the importance of selective targeting of M2-like macrophages in a therapeutic context (110).

Therapeutic blocking of the CCL2/CCR2 axis inhibits the recruitment of inflammatory monocytes and the infiltration and M2 polarization of TAMs, resulting in the reversal of the immunosuppression status of the TME and activation of an antitumor CD8^+^ T-cell response (102). However, a phase 2 study of carlumab, a human monoclonal antibody against CCL2, showed that carlumab failed to inhibit tumor growth since tumor cells compensatory increased the expression of CCL2 (111). Dual antagonists targeting both chemokine receptors simultaneously might be a strategy that could lead to a more effective TAM targeting. Chemokine receptors targeting agents need to be chosen accurately so as not to affect the recruitment of other immune cells such as natural killer (NK) cells and T cells.

CD47 has been proven to protect host cells from macrophage-mediated destruction by binding to signal regulatory protein (SIRP) 1α expressed on the surface of macrophages (112). Tang Z et al. revealed that CD47 could suppress phagocytosis not only by engaging SIRPα but also by masking cell-intrinsic pro-phagocytic ligands on tumor cells as well (113). Therefore, antibodies blocking CD47 and SIRPα might become an effective therapeutic strategy. The anti-CD47 monoclonal antibody B6H12 has been proven to induce macrophage-mediated phagocytosis, suppress tumor growth, and augment the efficacy of chemotherapy in HCC (104). Neutralizing antibodies against CD47 can enhance macrophage-mediated phagocytosis and activate effector T cells (114). The anti-CD40 monoclonal antibody selicrelumab is another approach to reprogram TAMs to an M1-like phenotype and enhance phagocytosis, whose main mode of action may be the induction of increased tumor-specific antigen presentation via activation of antigen-presenting cells, resulting in the production of cytotoxic T cells directed against the tumor (115–117).

TAMRs, expressed in tumors and various immune cells, exhibit diverse roles in processes such as cell fate, proliferation, migration, and regulation of tissue homeostasis and inflammation (118). Since TAMRs on macrophages have tumor-promoting roles of promoting M2-like polarization and efferocytosis, it is possible that targeting TAMRs on macrophages will be an effective therapy for treating different types of cancers (119).

## 5 Discussion and conclusion

As one of the most frequent malignancies worldwide, HCC is a serious threat to the lives and health of our people. The occurrence and development of HCC is a complex, multistep, and multifactor process. The polarization of TAMs, an important part and main immune cell of the TME of HCC, is affected by multiple signaling pathways and surrounding cells. TAMs participate in HCC progression by affecting HCC proliferation, invasion, and migration; mediating drug resistance; promoting angiogenesis; being involved in the formation of an immunosuppressive microenvironment; and reprogramming metabolic patterns. Owing to the crucial role that TAMs play in HCC progression, a better understanding of how TAMs regulate HCC malignancy is essential for the development of more effective TAM-targeting HCC therapies. The development and manufacture of highly selective targeting drugs will help promote the further development of antitumor immunotherapy targeting TAMs to improve clinical benefits for HCC patients.

Current investigations of TAMs in HCC remain insufficient. Although the macrophage polarization theory simplified macrophage biology by the M1-like/M2-like classification, increasing single-cell transcriptomics studies have captured a more complicated phenotype of macrophages and revealed the heterogeneous and high plasticity of TAMs at the transcriptional level. Therefore, further studies combined with genomics, proteomics, and transcriptomics analyses in both HCC *in situ* and metastasis are suggested to provide a more detailed understanding of the subtypes of macrophages in the TME of HCC and their corresponding functions in HCC.

Despite the encouraging results of clinical studies on TAMs, targeting TAMs in HCC treatment still faces some challenges. Most knowledge on how TAMs affect the TME of HCC is based on animal models. Considering the heterogeneity of tumor progression and therapy responses between animal models and humans, it is essential to explore inhibitors targeting human TAMs as well as their influence on the immunosuppressive microenvironment of HCC patients in order to enhance the applications of targeting TAM therapy strategies and improve outcomes.

In this review, we summarized the origin and phenotypic polarization of TAMs, their impact and molecular mechanism, and their potential applications in therapy strategies for HCC patients. We suggest further studies that focus on 1) identifying the diversity markers of macrophages to classify TAM subtypes, 2) revealing the heterogeneity of HCC tumors as well as the corresponding functions of TAMs in different locations such as HCC *in situ* and metastasis, and 3) enhancing the specificity of the markers for identifying TAM phenotypes. Through a better understanding of TAMs, future pharmaceuticals targeting TAMs in the specific immune environment of HCC combined with traditional immune therapy would provide a safer and more efficient treatment strategy for HCC patients to prolong survival and improve prognosis.

## Author contributions

YZ: Writing – original draft. GH: Writing – original draft. JG: Writing – original draft. ZC: Resources, Writing – original draft. JW: Supervision, Writing – review & editing.
